# The impact of female BMI on sperm DNA damage repair ability of oocytes and early embryonic development potential in intracytoplasmic sperm injection cycles

**DOI:** 10.3389/fendo.2023.1168010

**Published:** 2023-09-12

**Authors:** Yuqing Jiang, Zhaoyang Shen, Jianmin Xu, Jing Zhu, Huan Wang, Wenhui Chen, Yingpu Sun, Qingling Yang

**Affiliations:** ^1^ Center for Reproductive Medicine, The First Affiliated Hospital of Zhengzhou University, Zhengzhou, China; ^2^ Henan Key Laboratory of Reproduction and Genetics, The First Affiliated Hospital of Zhengzhou University, Zhengzhou, China; ^3^ Henan Provincial Obstetrical and Gynecological Diseases (Reproductive Medicine) Clinical Research Center, The First Affiliated Hospital of Zhengzhou University, Zhengzhou, China

**Keywords:** sperm DNA fragmentation index (DFI), overweight/obese, embryo development, fertilization, intracytoplasmic sperm injection

## Abstract

**Background:**

Obesity adversely influences the quality of oocytes and embryos and can affect DNA repair in embryos, leading to reproductive issues. However, the effects of body mass index (BMI) on DNA repair ability in oocytes during intracytoplasmic sperm injection (ICSI) cycles have not yet been investigated. Therefore, this retrospective study aimed to analyze the influence of sperm DNA damage on embryo development and reproductive outcomes in overweight/obese and normal-weight women in ICSI cycles.

**Methods:**

A total of 1,141 patients who received the first fresh ICSI cycle treatments were recruited from July 2017 to July 2021. Based on the BMI of the women, all patients were divided into normal weight (18.5≤BMI<25 kg/m^2^; n=824; 72.22%) and overweight/obese (BMI≥25 kg/m^2^; n=317; 27.78%) groups. Furthermore, according to the sperm DNA fragmentation index (DFI), these two groups were subdivided into two subgroups: DFI<30% and DFI≥30%.

**Results:**

In the normal-weight women group, the embryonic development and reproductive outcomes of ICSI cycles were not statistically different between the two subgroups (DFI<30% and DFI≥30%). However, in the overweight/obese women group, couples with a sperm DFI≥30% had a significantly lower fertilization rate (76% vs. 72.7%; p=0.027), cleavage rate (98.7% vs. 97.2%; p=0.006), and high-quality embryo rate (67.8% vs. 62.6%; p=0.006) than couples with a sperm DFI<30%.

**Conclusion:**

When injected sperm with high DFI into the oocytes of overweight/obese women, resulting in lower fertilization, cleavage, and high-quality embryo rates in ICSI cycles, and the decreased early developmental potential of embryos from overweight/obese patients may be caused by the diminished capacity of oocytes to repair sperm DNA damage.

## Introduction

Infertility is a public health issue, estimated to affect at least 186 million people worldwide (1). Approximately half of all infertility cases are caused by male factors (2), and 20% of these cases are attributed solely to male factors (3). Traditional semen parameters (volume, count, motility, and morphology) do not always predict male fertility (4). Semen parameters may be normal in approximately 15% of infertile men (5). The latest version of the WHO laboratory manual has acknowledged the limitations of the basal parameters of semen in predicting adverse reproductive outcomes, emphasized the significance of testing sperm function, and recommended sperm DNA fragmentation assessment in specific clinical settings, especially in cases of unexplained infertility (6).

Maintaining the integrity of germ cell genomes is essential for embryonic development and correct transmission of genetic information across generations (7). Oxidative stress and defects of sperm chromatin in the testes are the two primary factors causing sperm DNA breaks (8). In recent years, the DNA fragmentation index (DFI) has been considered a significant indicator for assessing the quality of semen, serving as a marker for DNA damage, and reflecting the integrity and degree of damage to sperm DNA (9). However, the correlation between DFI and fertility remains inconclusive and controversial to some extent. Evidence from two meta-analyses suggested that a higher DFI has negative consequences on the quality of embryos and leads to lower pregnancy rates and a higher rate of abortion during *in vitro* fertilization/intracytoplasmic sperm injection (IVF/ICSI) treatment (10, 11). In contrast, several other studies showed no significant differences in laboratory embryonic development and reproductive outcomes among different DFI groups during IVF or ICSI cycles (12, 13). The reported controversial effects of DFI on IVF or ICSI may be due to the failure of these studies to consider that the influence of DNA damage on fertility outcomes is affected by the integrity of sperm chromatin as well as oocyte repair ability and efficiency (14, 15).

Obesity adversely influences the quality of oocytes and embryos (16, 17) and can affect DNA repair in mouse embryos, leading to reproductive issues, such as decreased conception and loss of early pregnancy (18). With the development of assisted reproductive technology (ART), ICSI has become more prevalent. In most cases, a sperm with normal motility and morphology was chosen and injected into the oocyte during the ICSI cycle, bypassing the natural selection process of spermatozoa (19). Studies on sperm DNA damage affecting fertility outcomes during IVF/ICSI cycles have shown that a high level of DNA damage significantly affected clinical outcomes after IVF cycles, but not ICSI cycles (20–22). Therefore, if ICSI is selected as a method of fertilization, it can reduce the impact of DNA damage and compensate for the loss of sperm chromatin integrity, thus affecting the outcomes of ART. However, the effects of body mass index (BMI) on DNA repair ability in oocytes during ICSI cycles have not yet been investigated. We hypothesized that oocytes from overweight/obese women are less likely to repair sperm DNA damage, resulting in decreased embryo quality and even poor reproductive outcomes. Therefore, this retrospective study aimed to analyze the influence of sperm DNA damage on embryo development and reproductive outcomes in overweight/obese and normal-weight women in ICSI cycles.

## Material and methods

### Patient screening and study designs

We obtained data from the Reproductive Medical Center of the First Affiliated Hospital of Zhengzhou University using the Clinical Reproductive Medicine Management System/Electronic Medical Record Cohort Database. The First Affiliated Hospital of Zhengzhou University’s Institutional Review Board approved this study. A total of 1,141 patients who received the first fresh ICSI cycle treatments were recruited from July 2017 to July 2021. Inclusion criteria were: (1) all couples had normal chromosome karyotypes; (2) semen was obtained by ejaculation; (3) basal maternal follicle-stimulating hormone (FSH) level less than 12 mIU/mL; (4) females aged less than 40 years; and (5) treatment with a long follicular phase gonadotropin-releasing hormone (GnRH) agonist. The exclusion criteria were: (1) preimplantation genetic diagnosis/preimplantation genetic screening (PGD/PGS) cycles; (2) donor cycles; (3) women with polycystic ovary syndrome (PCOS); (4) diseases affecting pregnancy such as endometriosis, adenomyosis, uterine malformations, premature ovarian insufficiency (POI), and premature ovarian failure (POF); (5) couples in which one partner had significant endocrinology or metabolic dysfunctions such as thyroid dysfunction, hypertension, pelvic tuberculosis, diabetes, prolactinoma, hyperprolactinemia (HPRL), were also excluded before the study.

**Figure 1:**
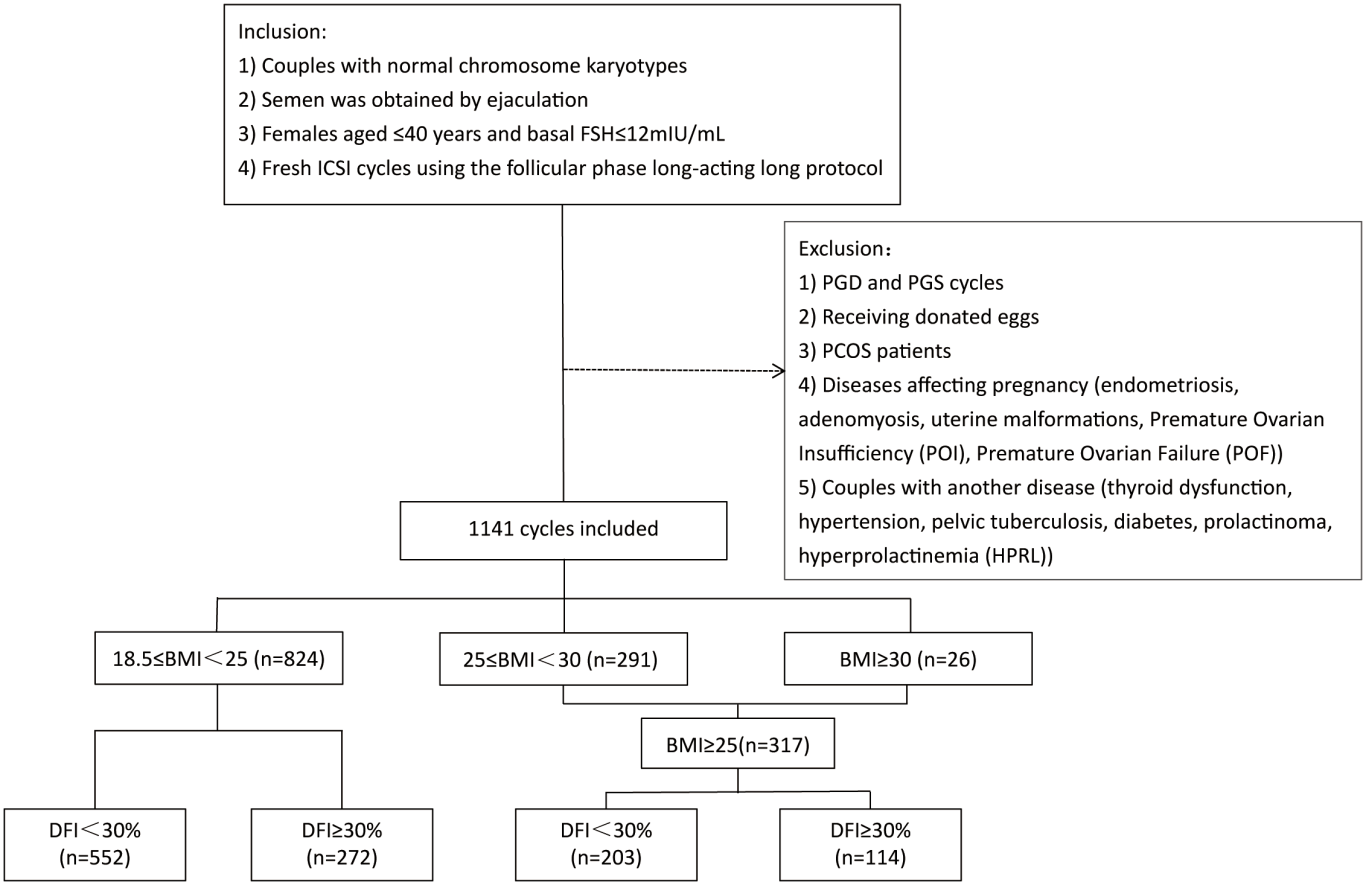
Detailed study flowchart and data processing procedure.

We included too few patients who met the World Health Organization (WHO) criteria for obesity; therefore, we combined obese patients (BMI≥30 kg/m2; n=26; 2.28%) and overweight patients (25≤BMI<30 kg/m2; n=291; 25.5%) into one group. All patients were divided into normal weight (18.5≤BMI<25 kg/m2; n=824; 72.22%) and overweight/obese (BMI≥25 kg/m2; n=317; 27.78%) groups. Furthermore, according to sperm DFI, these two groups were divided into two subgroups: DFI<30% and DFI≥30%. **Figure 1** shows the study flowchart and data processing procedure. Laboratory and pregnancy outcomes were analyzed in this study.

### Routine semen analysis and sperm DNA fragmentation index

After 3–7 days of sexual abstinence, semen samples were collected by masturbation on the day of oocyte recovery and one day before. According to the WHO laboratory manual, semen parameters, including concentration, motility, and morphology of sperm, were evaluated and combined with sperm preparation techniques for spermatozoa retention. DFI was detected using the standardized test of sperm chromatin structure analysis (SCSA). TNE solution [0.01 M Tris-HCl, 0.15 M NaCl, and 1 mM EDTA (pH 7.4)] was used to dilute the semen samples to a concentration of (0.5–1.0)×10^6^/mL. At the locations of strand breaks, DNA was denatured with an acid-detergent buffer [0.1% Triton X-100, 0.15 M NaCl, and 0.08 N HCl (pH 1.2)] and then stained using acridine orange solution (AO; pH 6.0). The fluorescence signals of 5,000–10,000 individual sperms were assessed by flow cytometry (BD FACS Canto II) to calculate the DFI by dividing the number of red spermatozoa by the total number of red and green spermatozoa. The fluorescence of different colors reflects the integrity and degree of damage to sperm DNA, which is shown as red DNA single-strand and green double-strand breaks.

### Intracytoplasmic sperm injection procedures

The follicular phase long-acting long protocol was used to stimulate the follicles. Intramuscular injections of the gonadotropin-releasing hormone agonist (GnRH; Diphereline, 3.75 mg; Beaufour-Ipsen, Dreux, France) were administered on the second and third menstrual days, and Gn was started 28 days later after they had achieved the downregulation criterion (no follicles >3–5 mm in diameter by ultrasound, no functional cyst; serum FSH<5 IU/L, estradiol (E2)<30 μg/mL, progesterone (P)<1 ng/mL, and luteinizing hormone (LH)<5 IU/L). The initial gonadotropin dose was administered based on female BMI, age, number of antral follicles, and reaction of the ovary to stimulation. To perform controlled ovarian stimulation, the dosage of Gn was adjusted according to the speed of follicular growth and the level of serum estrogen (23). When two or more follicles were 17 mm in diameter by ultrasound examination, recombinant human chorionic gonadotropin (hCG, Merck Serono, Italy) was administered to initiate the ovulation process. The cumulus oocyte complexes (COCs) were retrieved 36–37 h after the hCG trigger and placed right away in G-IVF Plus medium (Vitrolife Sweden AB, Goteborg, Sweden), where they were cultivated for 1-2 h at 37°C and in the presence of 6% CO_2_. By repeatedly aspirating with a 150-μm-diameter fine needle (Sunlight, FL, USA) with hyaluronidase (Vitrolife) digestion, the granulosa cells surrounding the COCs were eliminated. Then, mature oocytes were cultured in G-IVF Plus for 1–2 h for ICSI. Sperm was selected and injected into the oocytes in a micro-injection buffered medium. After injection, the oocytes were cultivated at 37°C and with 6% CO2 in a 50-μL G-1 Plus (Vitrolife) droplet covered with paraffin oil (Vitrolife) (24).

### Embryo culture

Approximately 16–18 h after ICSI, the embryos after fertilization were transferred to a sequential culture medium (Vitrolife, Sweden) in a 37°C incubator with an environment of 6% CO_2_ and 5% O_2_. Cleavage-stage embryos or blastocysts were transplanted according to the patient’s physical condition and embryo quality. The quality of cleavage-stage embryos and blastocysts was graded using a previously described grading system (25). The embryo transfer cycles in this study included only fresh cycles.

### Evaluation of pregnancy outcomes

On days 14 and 18 following embryo transfer, a positive serum human chorionic gonadotropin (hCG) test was defined as biochemical pregnancy. Thirty-five days after transplantation, ultrasonography was performed to detect the gestational sac to assess the clinical pregnancy. Clinical pregnancy loss before 28 weeks was considered an abortion.

### Clinical parameter definition

The implantation rate was calculated by dividing the number of gestational sacs with fetal heartbeats by the number of transplanted embryos. The pregnancy rate was defined as the number of pregnant women divided by the number of transplant women. Live birth rate was defined as the ratio of live births to transferred embryos. The clinical miscarriage rate is the number of couples with abortions divided by the number of clinical pregnancies.

### Statistical analysis

All fundamental aspects of women and men were displayed as continuous variables, and their characteristics were summarized as mean ± standard deviation. Moreover, we compared the variance between the two groups using an independent-samples t-test; categorical variables such as fertilization rate, cleavage rate, high-quality embryo rate, blastocyst formation rate, high-quality blastocyst rate, implantation rate, pregnancy rate, live birth rate, and abortion rate were characterized in terms of frequency and percentage, n (%), and to contrast proportions between groups, the chi-square test was employed. All data were analyzed using the Statistical Package for Social Sciences (SPSS) (Version 25.0, IBM, Armonk, NY). P<0.05 signified statistical significance.

## Results

### Characteristics of the study population

This research enrolled 1141 participants. Patient general characteristics as well as information on ovulation induction across BMI groups are shown in **Table 1**. In the normal weight (18.5≤BMI<25 kg/m^2^) group, basal levels of FSH (p<0.001) and E2 (p=0.006) were higher than those in the overweight/obese (BMI≥25 kg/m^2^) group. Both groups were within the normal range, although the differences were statistically significant. As shown in **Table 2**, the baseline characteristics (couple BMI, age, maternal basal FSH, E2, P, LH, and anti-Müllerian hormone (AMH) levels, and the number of matured oocytes) did not differ between the different subgroups within the two BMI groups.

**Table 1:** General characteristics of patients across two BMI groups.

	18.5 ≤ BMI < 25(n=824)	BMI ≥ 25(n=317)	P value
Maternal BMI (kg/m2)	21.78 ± 1.68	27.21 ± 1.83	<0.001
Maternal age (y)	30.97 ± 4.21	31.15 ± 4.35	0.531
Basal FSH	6.75 ± 2.09	6.26 ± 1.67	<0.001
Basal E2	42.39 ± 25.70	37.88 ± 22.42	0.006
Basal P	0.47 ± 1.03	0.38 ± 0.66	0.125
Basal LH	5.33 ± 3.08	5.21 ± 3.61	0.577
AMH	3.48 ± 2.59	3.62 ± 2.66	0.444
Paternal BMI (kg/m2)	25.48 ± 4.08	25.43 ± 3.71	0.831
Paternal age(y)	32.03 ± 5.25	32.29 ± 4.99	0.452

Data are presented as mean ± standard deviation (x ± SD). BMI, body mass index; FSH, follicle-stimulating hormone; E2, estradiol; P, progestogen; LH, luteinizing hormone; AMH, anti-Müllerian hormone.

**Table 2:** The baseline characteristics of the two subgroups within BMI groups.

	18.5 ≤ BMI < 25 (n=824)			BMI ≥ 25 (n=317)		
	DFI<30 (n=552)	DFI≥30 (n=272)	P	DFI<30 (n=203)	DFI≥30 (n=114)	P
Maternal BMI (kg/m2)	21.76 ± 1.68	21.81 ± 1.69	0.680	27.25 ± 1.96	27.14 ± 1.58	0.629
Maternal age(y)	31.03 ± 4.00	30.86 ± 4.62	0.623	31.43 ± 4.38	30.64 ± 4.28	0.119
Basal FSH	6.79 ± 2.23	6.68 ± 1.78	0.460	6.26 ± 1.73	6.26 ± 1.56	0.991
Basal E2	42.72 ± 27.00	41.73 ± 22.85	0.603	39.26 ± 25.79	35.44 ± 14.38	0.091
Basal P	0.50 ± 1.18	0.42 ± 0.60	0.278	0.39 ± 0.78	0.35 ± 0.35	0.568
Basal LH	5.19 ± 3.05	5.63 ± 3.14	0.057	5.45 ± 3.84	4.77 ± 3.12	0.089
AMH	3.49 ± 2.75	3.47 ± 2.24	0.885	3.79 ± 3.00	3.31 ± 1.90	0.087
Paternal BMI (kg/m2)	25.59 ± 4.14	25.26 ± 3.96	0.279	25.16 ± 3.67	25.88 ± 3.74	0.098
Paternal age(y)	31.91 ± 4.92	32.27 ± 5.88	0.388	32.21 ± 4.86	32.43 ± 5.23	0.710
Paternal DFI	15.90 ± 7.78	44.39 ± 12.33	<0.001	15.06 ± 7.07	44.99 ± 12.48	<0.001
Retrieved oocytes (n)	13.17 ± 6.05	13.24 ± 5.73	0.863	13.98 ± 7.00	14.61 ± 6.71	0.431
Mature oocytes (n)	10.78 ± 5.22	11.14 ± 5.24	0.359	11.37 ± 5.90	12.23 ± 5.64	0.208

Data are presented as mean ± standard deviation (x ± SD). BMI, body mass index; FSH, follicle-stimulating hormone; E2, estradiol; P, progestogen; LH, luteinizing hormone; AMH, anti-Müllerian hormone.

### Cycles outcomes

**Table 3** shows the results of laboratory and clinical outcome analyses for each subgroup. For the normal weight (18.5≤BMI<25 kg/m^2^) group, there were no significant differences between the two subgroups (DFI < 30% and DFI ≥ 30%). When maternal BMI was outside the normal range (BMI≥25 kg/m^2^), no statistical difference was noted between the two DFI level groups in terms of blastocyst formation rate, high-quality blastocyst rate, number of embryos transferred, implantation rate, clinical pregnancy rate, live birth rate, and abortion rate. However, the DFI≥30% group had a substantially lower fertilization rate (76% vs. 72.7%; p=0.027), cleavage rate (98.7% vs. 97.2%; p=0.006), and high-quality embryo rate (67.8% vs. 62.6%; p=0.006) than the DFI<30% group.

**Table 3:** Laboratory and clinical outcomes comparison in patients with different sperm DFI in each BMI group.

Laboratory and clinical outcomes	18.5 ≤ BMI < 25 (n=824)			BMI ≥ 25 (n=317)		
	DFI<30 (n=552)	DFI≥30 (n=272)	P	DFI<30 (n=203)	DFI≥30 (n=114)	P
Fertilization rate	4442/5951 (74.6)	2217/3029 (73.2)	0.138	1754/2308 (76.0)	1014/1394 (72.7)	0.027
Cleavage rate	4402/4442 (99.1)	2188/2217 (98.7)	0.122	1731/1754 (98.7)	986/1014 (97.2)	0.006
High-quality embryo rate	2947/4402 (66.9)	1512/2188 (69.1)	0.078	1173/1731 (67.8)	617/986 (62.6)	0.006
Blastocyst formation rate	1517/2729 (55.6)	779/1374 (56.7)	0.500	676/1170 (57.8)	343/613 (56.0)	0.460
High-quality blastocyst rate	624/1517 (41.1)	335/779 (43.0)	0.390	284/676 (42.0)	143/343 (41.7)	0.922
Number of embryos transferred	1.63 ± 0.48	1.62 ± 0.49	0.806	1.12 ± 0.824	1.11 ± 0.849	0.966
Implantation rate	315/666 (47.3)	154/322 (47.8)	0.876	107/227 (47.1)	67/127 (52.8)	0.310
Pregnancy rate	245/409 (59.9)	123/199 (61.8)	0.652	88/145 (60.7)	52/80 (65.0)	0.523
Live birth rate	271/666 (40.7)	138/322 (42.9)	0.517	87/227 (38.3)	56/127 (44.1)	0.289
Clinical miscarriage rate	31/245 (12.7)	11/123 (8.9)	0.291	12/88 (13.6)	8/52 (15.4)	0.775

Data are presented as mean ± standard deviation (x ± SD) or the frequency(percentage).

In addition, we compared clinical and laboratory outcomes between DFI<30% and DFI≥30% cycles to analyze differences between normal, overweight, and obese women.(**Supplementary Tables 1**, **2** in **Supplementary Materials**). The results are broadly consistent with the results of the above analysis, showing that in the DFI≥30% group, cleavage rate (98.7% vs. 97.1%; p=0.006), and high-quality embryo rate (69.1% vs. 62.3%; p=0.001) had a significant difference between 18.5≤BMI<25 kg/m^2^ and 25≤BMI<30 kg/m^2^ groups, but not DFI < 30% group.

## Discussion

The current study indicated that sperm DNA fragmentation had no effect on embryo development or reproductive outcomes in normal-weight women. However, in overweight/obese women, lower fertilization rate, cleavage rate, and high-quality embryo rate were observed in DFI≥30% cycles than in DFI<30% cycles. These findings are congruent with prior research on IVF cycles at our center (26), which revealed that in patients with normal weight, there was no statistically significant difference in the different levels of sperm fragmentation in terms of embryo quality and reproductive outcomes. However, in overweight patients, the high-fragmentation group had much lower rates of fertilization, blastocyst development, and high-quality blastocysts than the low-fragmentation group. Simultaneously, echoing our results, the clinical outcomes were also not statistically different between the two groups with different DFI. The findings of the present study support our hypothesis and suggest that a higher DFI has an adverse influence on early embryonic development in overweight/obese but not normal-weight women in ICSI cycles.

Being overweight or obese in women is inherently associated with reduced reproductive outcomes (27). Previous studies have shown that obesity could impair the maturation of oocytes, reduce oocyte polarity, disrupt mitochondrial dynamics as well as spindle morphology during meiosis (17, 28), and obesity may increase reactive oxygen species (ROS), which may subsequently result in DNA damage (29, 30). In addition, several studies have revealed that obesity negatively influences embryonic development by altering the DNA methylation levels of genes involved in metabolism (31). Animal experiments have shown that in early mouse embryos, obesity can also impair the establishment of pronuclear epigenetic asymmetry in zygotes and activate autophagy/apoptosis (18, 32). In addition, sperm cannot repair DNA damage (33, 34). DNA damage needs to be repaired by oocytes after fertilization (35). The cytoplasm and genome quality of oocytes play a vital role in the repair of sperm DNA fragmentation (36, 37). However, obesity alters the mRNAs and proteins levels of DNA repair response genes, resulting in dysregulated DNA repair proteins and processes (38, 39). An animal genome-wide analysis demonstrated that the levels of gene expression that are involved in DNA damage and repair were altered in obese embryos, including H2A.X, Rad51, and Tex15. Furthermore, the rates of 4-cell and morula/blastocyst formation were dramatically reduced during embryo development (18). Taken together, obesity in women may increase DNA damage in oocytes and impede repair, leading to genetic instability. Therefore, these deficiencies result in a reduced ability and efficiency to repair sperm DNA damage in oocytes and early embryos in obese women, which ultimately compromises reproductive outcomes.

Recent research on sperm selection during ICSI treatment showed that double-strand DNA break (DSB) has an enzymatic and controlled origin, which does not correlate with sperm motility (40). Even if a sperm with normal motility and morphology is selected and injected directly into the cytoplasm of a mature oocyte during the ICSI cycle, the selected spermatozoa may have a defective genome with a high DFI (41). Stephanie et al. reported a negative correlation between DFI and fertilization rate in ICSI (42). Another study showed that using sperm with highly fragmented DNA in the ICSI cycle leads to a low rate of fertilization (43). In addition to highly abnormal sperm chromatin (44), failure of ICSI fertilization may also be caused by oocytes, including oocyte chromosomal DNA fragmentation, oocyte cytoplasmic and nuclear maturation, or activation failure, and thereby fail to decondense sperm nuclei by oocytes (45–47). Therefore, when the quality of oocytes and the capacity to repair the damage of sperm DNA in overweight patients is decreased and sperm with highly fragmented DNA is used for injection, the fertilization rate may be reduced. This was also confirmed in the current investigation, which showed that overweight/obese women with high DFI couples had reduced fertilization rate.

Evidence suggests that oocytes cannot effectively repair sperm DNA damage in zygotes, which may affect subsequent post-fertilization steps including embryonic development and clinical outcomes (48). In addition to female obesity, other unfavorable conditions, such as advanced maternal age and PCOS, similarly decrease oocyte quality and the ability to repair sperm DNA damage. Khalafalla et al. showed that during ICSI cycles, when sperm samples with a higher DFI were injected, women with advanced maternal age had considerably lower clinical pregnancy rates and live birth rates compared to younger women (49). Another study also indicated that oocyte capacity to repair sperm DNA damage was decreased in older women, resulting in poor embryo quality, lower implantation and pregnancy rates, and a higher miscarriage rate (50). In addition, the good-quality blastocyst rate was much lower due to the diminished capacity to repair DNA damage in oocytes among PCOS patients, while the sperm DFI was high (51). These results suggest that the quality of oocytes and the capacity to repair the sperm DNA damage are important factors affecting the outcome of ART when the sperm DFI of the male partner is high.

In summary, the embryonic development and reproductive outcomes of ICSI cycles in normal-weight women were not significantly different among the different DFI groups. However, oocytes from overweight/obese women were injected with high-DFI sperm, resulting in lower fertilization, cleavage, and high-quality embryo rates in ICSI cycles. The decreased capacity of oocytes to repair sperm DNA damage in overweight/obese women is an important impact of these results. The phenomenon of similar pregnancy outcomes between different DFI levels in overweight/obese patients may be explained by the fact that selecting high-quality embryos or blastocysts for transfer may avoid the negative impacts of sperm DNA fragmentation on clinical outcomes.

The results of this study have an important guiding significance for recommending weight loss in overweight/obese women before ART. In addition, because the repair ability of human oocytes, especially the poor quality of oocytes, is not sufficient to overcome paternal sperm DNA damage, improving the quality of spermatozoa is also important. Some suggestions were made to reduce sperm DFI, such as shortening the male abstinence time as appropriate, changing unhealthy lifestyle habits (smoking and alcohol drinking) and diet (13), and supplementing with antioxidants as recommended by the physician (52).

## Conclusions

In conclusion, when injected sperm with high DFI into oocytes of overweight/obese women, resulting in lower fertilization, cleavage, and high-quality embryo rates in ICSI cycles, and the decreased early developmental potential of embryos from overweight/obese patients may be caused by the diminished capacity of oocytes to repair sperm DNA damage.

## Data availability statement

The raw data supporting the conclusions of this article will be made available by the authors, without undue reservation.

## Ethics statement

The studies involving human participants were reviewed and approved by The First Affiliated Hospital of Zhengzhou University’s Institutional Review Board. The patients/participants provided their written informed consent to participate in this study.

## Author contributions

QY and YS contributed to the study conception and design. QY and YJ were responsible for the data analysis and article drafting. ZS and JX participated in data collection. JZ, HW, and WC took part in the discussion of the results. All authors contributed to the article and approved the submitted version.

## References

[1] Inhorn MC Patrizio P. Infertility around the globe: new thinking on gender, reproductive technologies and global movements in the 21st century. Hum Reprod Update (2015) 21(4):411–26. doi: 10.1093/humupd/dmv016 25801630

[2] Lotti F Maggi M. Sexual dysfunction and Male infertility. Nat Rev Urol (2018) 15(5):287–307. doi: 10.1038/nrurol.2018.20 29532805

[3] Thonneau P Marchand S Tallec A Ferial ML Ducot B Lansac J. Incidence and main causes of infertility in a resident population (1,850,000) of three French regions (1988-1989). Hum Reprod (Oxford England) (1991) 6(6):811–6. doi: 10.1093/oxfordjournals.humrep.a137433 1757519

[4] Findeklee S Radosa JC Radosa MP Hammadeh ME. Correlation between total sperm count and sperm motility and pregnancy rate in couples undergoing intrauterine insemination. Sci Rep (2020) 10(1):7555. doi: 10.1038/s41598-020-64578-0 32371917 PMC7200727

[5] Agarwal A Allamaneni SS. Sperm DNA damage assessment: a test whose time has come. Fertility sterility (2005) 84(4):850–3. doi: 10.1016/j.fertnstert.2005.03.080 16213833

[6] Baldini D Ferri D Baldini GM Lot D Catino A Vizziello D. Sperm selection for icsi: do we have a winner? Cells (2021) 10(12). doi: 10.3390/cells10123566 PMC8700516 34944074

[7] Leem J Bai GY Oh JS. The capacity to repair sperm DNA damage in zygotes is enhanced by inhibiting Wip1 activity. Front Cell Dev Biol (2022) 10:841327. doi: 10.3389/fcell.2022.841327 35478962 PMC9037036

[8] Muratori M Tamburrino L Marchiani S Cambi M Olivito B Azzari C. Investigation on the origin of sperm DNA fragmentation: role of apoptosis, immaturity and oxidative stress. Mol Med (Cambridge Mass) (2015) 21(1):109–22. doi: 10.2119/molmed.2014.00158 PMC4461587 25786204

[9] Sakkas D Alvarez JG. Sperm DNA fragmentation: mechanisms of origin, impact on reproductive outcome, and analysis. Fertil Steril (2010) 93(4):1027–36. doi: 10.1016/j.fertnstert.2009.10.046 20080235

[10] Deng C Li T Xie Y Guo Y Yang QY Liang X. Sperm DNA fragmentation index influences assisted reproductive technology outcome: a systematic review and meta-analysis combined with a retrospective cohort study. Andrologia (2019) 51(6):e13263. doi: 10.1111/and.13263 30838696

[11] Simon L Zini A Dyachenko A Ciampi A Carrell DT. A systematic review and meta-analysis to determine the effect of sperm DNA damage on in vitro fertilization and intracytoplasmic sperm injection outcome. Asian J Androlog (2017) 19(1):80–90. doi: 10.4103/1008-682x.182822 PMC5227680 27345006

[12] Green KA Patounakis G Dougherty MP Werner MD Scott RT Jr. Franasiak JM. Sperm DNA fragmentation on the day of fertilization is not associated with embryologic or clinical outcomes after Ivf/Icsi. J Assist Reprod Genet (2020) 37(1):71–6. doi: 10.1007/s10815-019-01632-5 PMC7000566 31755002

[13] Yang H Li G Jin H Guo Y Sun Y. The effect of sperm DNA fragmentation index on assisted reproductive technology outcomes and its relationship with semen parameters and lifestyle. Trans Androlog Urol (2019) 8(4):356–65. doi: 10.21037/tau.2019.06.22 PMC6732090 31555559

[14] Meseguer M Santiso R Garrido N García-Herrero S Remohí J Fernandez JL. Effect of sperm DNA fragmentation on pregnancy outcome depends on oocyte quality. Fertil Steril (2011) 95(1):124–8. doi: 10.1016/j.fertnstert.2010.05.055 20643402

[15] Marchetti F Essers J Kanaar R Wyrobek AJ. Disruption of maternal DNA repair increases sperm-derived chromosomal aberrations. Proc Natl Acad Sci USA (2007) 104(45):17725–9. doi: 10.1073/pnas.0705257104 PMC2077046 17978187

[16] Metwally M Cutting R Tipton A Skull J Ledger WL Li TC. Effect of increased body mass index on oocyte and embryo quality in ivf patients. Reprod Biomed Online (2007) 15(5):532–8. doi: 10.1016/s1472-6483(10)60385-9 18044034

[17] Hou YJ Zhu CC Duan X Liu HL Wang Q Sun SC. Both diet and gene mutation induced obesity affect oocyte quality in mice. Sci Rep (2016) 6:18858. doi: 10.1038/srep18858 26732298 PMC4702149

[18] Pan MH Zhu CC Ju JQ Xu Y Luo SM Sun SC. Single-cell transcriptome analysis reveals that maternal obesity affects DNA repair, histone methylation, and autophagy level in mouse embryos. J Cell Physiol (2021) 236(7):4944–53. doi: 10.1002/jcp.30201 33368268

[19] Rubino P Viganò P Luddi A Piomboni P. The icsi procedure from past to future: a systematic review of the more controversial aspects. Hum Reprod Update (2016) 22(2):194–227. doi: 10.1093/humupd/dmv050 26586241

[20] Zhao J Zhang Q Wang Y Li Y. Whether sperm deoxyribonucleic acid fragmentation has an effect on pregnancy and miscarriage after *In vitro* Fertilization/Intracytoplasmic sperm injection: a systematic review and meta-analysis. Fertil Steril (2014) 102(4):998–1005.e8. doi: 10.1016/j.fertnstert.2014.06.033 25190048

[21] Ribas-Maynou J Yeste M Becerra-Tomás N Aston KI James ER Salas-Huetos A. Clinical implications of sperm DNA damage in ivf and icsi: updated systematic review and meta-analysis. Biol Rev Cambridge Philos Soc (2021) 96(4):1284–300. doi: 10.1111/brv.12700 33644978

[22] Liang X Mao Y Wang Y Liu S Yan J. Female age affects the utility of sperm DNA fragmentation in predicting ivf and icsi outcomes. Reprod Biomed Online (2019) 39(6):955–62. doi: 10.1016/j.rbmo.2019.09.013 31753711

[23] Li G Wu Y Niu W Xu J Hu L Shi H. Analysis of the number of euploid embryos in preimplantation genetic testing cycles with early-follicular phase long-acting gonadotropin-releasing hormone agonist long protocol. Front Endocrinol (2020) 11:424. doi: 10.3389/fendo.2020.00424 PMC7386196 32793112

[24] Xu Z Yao G Niu W Fan H Ma X Shi S. Calcium ionophore (A23187) rescues the activation of unfertilized oocytes after intracytoplasmic sperm injection and chromosome analysis of blastocyst after activation. Front Endocrinol (2021) 12:692082. doi: 10.3389/fendo.2021.692082 PMC8320372 34335469

[25] Gardner DK Lane M Stevens J Schlenker T Schoolcraft WB. Blastocyst score affects implantation and pregnancy outcome: towards a single blastocyst transfer. Fertil Steril (2000) 73(6):1155–8. doi: 10.1016/s0015-0282(00)00518-5 10856474

[26] Li H Wang H Zhu J Xu J Jiang Y Chen W. Decreased DNA repair ability: a mechanism for low early embryonic development potential of oocytes from overweight patients after fertilization in ivf cycles. Front Endocrinol (2021) 12:756336. doi: 10.3389/fendo.2021.756336 PMC8651388 34887832

[27] Silvestris E de Pergola G Rosania R Loverro G. Obesity as disruptor of the female fertility. Reprod Biol Endocrinol RB&E (2018) 16(1):22. doi: 10.1186/s12958-018-0336-z 29523133 PMC5845358

[28] Broughton DE Moley KH. Obesity and female infertility: potential mediators of obesity's impact. Fertil Steril (2017) 107(4):840–7. doi: 10.1016/j.fertnstert.2017.01.017 28292619

[29] Kompella P Vasquez KM. Obesity and cancer: a mechanistic overview of metabolic changes in obesity that impact genetic instability. Mol Carcinog (2019) 58(9):1531–50. doi: 10.1002/mc.23048 PMC6692207 31168912

[30] de Mello AH Costa AB Engel JDG Rezin GT. Mitochondrial dysfunction in obesity. Life Sci (2018) 192:26–32. doi: 10.1016/j.lfs.2017.11.019 29155300

[31] Ou XH Zhu CC Sun SC. Effects of obesity and diabetes on the epigenetic modification of mammalian gametes. J Cell Physiol (2019) 234(6):7847–55. doi: 10.1002/jcp.27847 30536398

[32] Han L Ren C Li L Li X Ge J Wang H. Embryonic defects induced by maternal obesity in mice derive from Stella insufficiency in oocytes. Nat Genet (2018) 50(3):432–42. doi: 10.1038/s41588-018-0055-6 29459681

[33] Ward WS Coffey DS. DNA Packaging and organization in mammalian spermatozoa: comparison with somatic cells. Biol Reprod (1991) 44(4):569–74. doi: 10.1095/biolreprod44.4.569 2043729

[34] Baarends WM van der Laan R Grootegoed JA. DNA Repair mechanisms and gametogenesis. Reprod (Cambridge England) (2001) 121(1):31–9. doi: 10.1530/rep.0.1210031 11226027

[35] Ménézo Y Dale B Cohen M. DNA Damage and repair in human oocytes and embryos: a review. Zygote (Cambridge England) (2010) 18(4):357–65. doi: 10.1017/s0967199410000286 20663262

[36] Fernández-Díez C González-Rojo S Lombó M Herráez MP. Impact of sperm DNA damage and oocyte-repairing capacity on trout development. Reprod (Cambridge England) (2016) 152(1):57–67. doi: 10.1530/rep-16-0077 27071918

[37] García-Rodríguez A Gosálvez J Agarwal A Roy R Johnston S. DNA Damage and repair in human reproductive cells. Int J Mol Sci (2018) 20(1). doi: 10.3390/ijms20010031 PMC6337641 30577615

[38] D'Amico AM Vasquez KM. The multifaceted roles of DNA repair and replication proteins in aging and obesity. DNA Repair (2021) 99:103049. doi: 10.1016/j.dnarep.2021.103049 33529944 PMC7941874

[39] Ganesan S Nteeba J Madden JA Keating AF. Obesity alters phosphoramide mustard-induced ovarian DNA repair in mice. Biol Reprod (2017) 96(2):491–501. doi: 10.1095/biolreprod.116.143800 28203708 PMC6366544

[40] Lara-Cerrillo S Ribas-Maynou J Rosado-Iglesias C Lacruz-Ruiz T Benet J García-Peiró A. Sperm selection during icsi treatments reduces single- but not double-strand DNA break values compared to the semen sample. J Assist Reprod Genet (2021) 38(5):1187–96. doi: 10.1007/s10815-021-02129-w PMC8190426 33660206

[41] Evenson DP Larson KL Jost LK. Sperm chromatin structure assay: its clinical use for detecting sperm DNA fragmentation in Male infertility and comparisons with other techniques. J Androlog (2002) 23(1):25–43. doi: 10.1002/j.1939-4640.2002.tb02599.x 11780920

[42] Lopes S Sun JG Jurisicova A Meriano J Casper RF. Sperm deoxyribonucleic acid fragmentation is increased in poor-quality semen samples and correlates with failed fertilization in intracytoplasmic sperm injection. Fertil Steril (1998) 69(3):528–32. doi: 10.1016/s0015-0282(97)00536-0 9531891

[43] Sun JG Jurisicova A Casper RF. Detection of deoxyribonucleic acid fragmentation in human sperm: correlation with fertilization in vitro. Biol Reprod (1997) 56(3):602–7. doi: 10.1095/biolreprod56.3.602 9047003

[44] Sakkas D Urner F Bianchi PG Bizzaro D Wagner I Jaquenoud N. Sperm chromatin anomalies can influence decondensation after intracytoplasmic sperm injection. Hum Reprod (Oxford England) (1996) 11(4):837–43. doi: 10.1093/oxfordjournals.humrep.a019263 8671337

[45] Ferrer-Buitrago M Bonte D Dhaenens L Vermorgen S Lu Y De Sutter P. Assessment of the calcium releasing machinery in oocytes that failed to fertilize after conventional icsi and assisted oocyte activation. Reprod Biomed Online (2019) 38(4):497–507. doi: 10.1016/j.rbmo.2018.12.035 30745236

[46] Eppig JJ. Coordination of nuclear and cytoplasmic oocyte maturation in eutherian mammals. Reprod Fertil Dev (1996) 8(4):485–9. doi: 10.1071/rd9960485 8870074

[47] Racowsky C Prather AL Johnson MK Olvera SP Gelety TJ. Prematurely condensed chromosomes and meiotic abnormalities in unfertilized human oocytes after ovarian stimulation with and without gonadotropin-releasing hormone agonist. Fertil Steril (1997) 67(5):932–8. doi: 10.1016/s0015-0282(97)81410-0 9130903

[48] Tamburrino L Marchiani S Montoya M Elia Marino F Natali I Cambi M. Mechanisms and clinical correlates of sperm DNA damage. Asian J andrology (2012) 14(1):24–31. doi: 10.1038/aja.2011.59 PMC3735140 22138903

[49] Khalafalla K Majzoub A Elbardisi H Bhathella A Chaudhari A Agarwal A. The effect of sperm DNA fragmentation on intracytoplasmic sperm injection outcome. Androlog (2021) 53(10):e14180. doi: 10.1111/and.14180 34247427

[50] Setti AS Braga D Provenza RR Iaconelli A Jr. Borges E Jr. Oocyte ability to repair sperm DNA fragmentation: the impact of maternal age on intracytoplasmic sperm injection outcomes. Fertil Steril (2021) 116(1):123–9. doi: 10.1016/j.fertnstert.2020.10.045 33589137

[51] Wang H Li H Zhu J Xu J Jiang Y Chen W. The effect of sperm DNA fragmentation on in vitro fertilization outcomes for women with polycystic ovary syndrome. Front Endocrinol (2022) 13:822786. doi: 10.3389/fendo.2022.822786 PMC9196864 35712248

[52] Nassan FL Chavarro JE Tanrikut C. Diet and men's fertility: does diet affect sperm quality? Fertil Steril (2018) 110(4):570–7. doi: 10.1016/j.fertnstert.2018.05.025 30196939

