# Supplementary material for: The impact of female BMI on sperm DNA damage repair ability of oocytes and early embryonic development potential in intracytoplasmic sperm injection cycles
Source: Front Endocrinol (Lausanne). 2023 Sep 12;14:1168010. doi: 10.3389/fendo.2023.1168010 (PMC10534975; doi:10.3389/fendo.2023.1168010)
Supplement: Supplementary file 1 [file DataSheet_1.docx]

**Supplementary Materials**

**Supplementary Table1：**

The baseline characteristics of the subgroups within DFI groups.

|  | DFI < 30（n=755） | | | | DFI ≥ 30 （n=386） | | | |
| --- | --- | --- | --- | --- | --- | --- | --- | --- |
|  | 18.5 ≤ BMI < 25 (n=552) | 25 ≤ BMI < 30 (n=184) | BMI ≥30  (n=19) | P | 18.5 ≤ BMI < 25 (n=272) | 25 ≤ BMI < 30 (n=107) | BMI ≥30 (n=7) | P |
| Maternal BMI (kg/m2) | 21.76±1.68 | 26.79±1.36 | 31.70±1.14 | 0.001 | 21.81±1.69 | 26.89±1.27 | 30.99±0.30 | 0.001 |
| Maternal age(y) | 31.03±4.00 | 31.46±4.37 | 31.16±4.52 | 0.458 | 30.86±4.62 | 30.84±4.22 | 27.57±4.31 | 0.162 |
| Basal FSH | 6.79±2.23 | 6.27±1.73 | 6.21±1.75 | 0.010 | 6.676±1.78 | 6.278±1.56 | 6.01±1.61 | 0.090 |
| Basal E2 | 42.72±27.00 | 39.66±26.46 | 35.32±18.01 | 0.229 | 41.73±22.85 | 34.94±14.55 | 43.00±9.22 | 0.016 |
| Basal P | 0.50±1.18 | 0.40±0.81 | 0.34±0.31 | 0.479 | 0.42±0.60 | 0.35±0.36 | 0.37±0.28 | 0.526 |
| Basal LH | 5.19±3.056 | 5.44±3.82 | 5.56±4.13 | 0.617 | 5.63±3.14 | 4.64±3.00 | 6.68±4.46 | 0.013 |
| AMH | 3.49±2.75 | 3.87±3.078 | 3.02±2.01 | 0.208 | 3.466±2.24 | 3.30±1.92 | 3.44±1.63 | 0.805 |
| Paternal BMI (kg/m^2^) | 25.59±4.14 | 25.09±3.65 | 25.90±3.90 | 0.321 | 25.26±3.96 | 25.73±3.75 | 28.25±2.80 | 0.091 |
| Paternal age(y) | 31.91±4.93 | 32.30±4.90 | 31.37±4.50 | 0.559 | 32.27±5.88 | 32.55±5.31 | 30.57±3.60 | 0.653 |
| Paternal DFI | 15.90±7.78 | 15.30±7.13 | 12.78±6.19 | 0.158 | 44.39±12.33 | 44.86±12.47 | 47.13±13.45 | 0.812 |
| Retrieved oocytes (n) | 10.78±5.21 | 11.24±5.56 | 12.63±8.63 | 0.235 | 13.24±5.73 | 14.55±6.70 | 15.57±7.28 | 0.116 |
| Mature oocytes (n) | 8.047±4.52 | 8.44±4.83 | 10.58±8.27 | 0.053 | 8.15±4.44 | 8.94±4.64 | 8.14±5.98 | 0.306 |

Data are presented as mean ± standard deviation (x ± SD). BMI, body mass index; FSH, follicle-stimulating hormone; E2, estradiol; P, progestogen; LH, luteinizing hormone; AMH, anti-Müllerian hormone.

**Supplementary Table2：**

Laboratory and clinical outcome comparison in patients with different BMI in each sperm DFI group.

| Laboratory and clinical outcomes | DFI < 30（n=755） | | | | DFI ≥ 30（n=386） | | | |
| --- | --- | --- | --- | --- | --- | --- | --- | --- |
|  | 18.5 ≤ BMI < 25 (n=552) | 25 ≤ BMI < 30 (n=184) | BMI ≥30 (n=19) | P | 18.5 ≤ BMI < 25 (n=272) | 25 ≤ BMI < 30 (n=107) | BMI ≥30 (n=7) | P |
| Fertilization rate | 4442/5951  (74.6)^a^ | 1553/2068  (75.1)^a^ | 201/240  (83.8)^b^ | 0.006 | 2217/3029  (73.2)^a^ | 957/1294  (74.0)^a^ | 57/100  (57.0)^b^ | 0.001 |
| Cleavage rate | 4402/4442  (99.1) | 1533/1553  (98.7) | 198/201  (98.5) | 0.336 | 2188/2217  (98.7)^a^ | 929/957  (97.1)^b^ | 57/57  (100)^a,b^ | 0.004 |
| High-quality embryo rate | 2947/4402  (66.9) | 1030/1533  (67.2) | 143/198  (72.2) | 0.302 | 1512/2188  (69.1)^a^ | 579/929  (62.3)^b^ | 38/57  (66.7)^a,b^ | 0.001 |
| Blastocyst formation rate | 1517/2729  (55.6) | 588/1023  (57.5) | 88/147  (59.9) | 0.388 | 779/1374  (56.7) | 327/576  (56.8) | 16/37  (43.2) | 0.261 |
| High-quality blastocyst rate | 624/1517  (41.1)^a^ | 236/588  (40.1)^a^ | 48/88  (54.5)^b^ | 0.035 | 335/779  (43.0) | 133/327  (40.7) | 10/16  (62.5) | 0.208 |
| Number of embryos transferred | 1.63±0.48^a^ | 1.11±0.83^b^ | 1.16±0.83^b^ | ＜0.001 | 1.62±0.49^a^ | 1.11±0.85^b^ | 1.14±0.9^b^ | 0.001 |
| Implantation rate | 315/666  (47.3) | 93/205  (45.4) | 14/22  (63.6) | 0.264 | 154/322  (47.8) | 63/119  (52.9) | 4/8  (50) | 0.634 |
| Pregnancy rate | 245/409  (59.9) | 77/131  (58.8) | 11/14  (78.6) | 0.351 | 123/199  (61.8) | 48/75  (64.0) | 4/5  (80.0) | 0.683 |
| Live birth rate | 271/666  (40.7) | 75/205  (36.6) | 12/22  (54.5) | 0.216 | 138/322  (42.9) | 52/119  (43.7) | 4/8  (50.0) | 0.915 |
| Clinical miscarriage rate | 31/245  (12.7) | 11/77  (14.3) | 1/11  (9.1) | 0.866 | 11/123  (8.9) | 8/48  (16.7) | 0/4  (0.0) | 0.269 |

Data are presented as mean ± standard deviation (x ± SD) or the frequency(percentage).

There were significant differences between different letter labels of a/b
